# Concepts to Reveal Parvovirus–Nucleus Interactions

**DOI:** 10.3390/v13071306

**Published:** 2021-07-05

**Authors:** Salla Mattola, Satu Hakanen, Sami Salminen, Vesa Aho, Elina Mäntylä, Teemu O. Ihalainen, Michael Kann, Maija Vihinen-Ranta

**Affiliations:** 1 Department of Biological and Environmental Science, University of Jyvaskyla, 40500 Jyvaskyla, Finland; salla.m.mattola@jyu.fi (S.M.); satu.a.hakanen@jyu.fi (S.H.); Sami.j.salminen@jyu.fi (S.S.); Vesa.p.aho@jyu.fi (V.A.); 2 BioMediTech, Faculty of Medicine and Health Technology, Tampere University, 33520 Tampere, Finland; elina.mantyla@tuni.fi (E.M.); teemu.ihalainen@tuni.fi (T.O.I.); 3 Department of Infectious Diseases, Institute of Biomedicine, Sahlgrenska Academy, University of Gothenburg, 41390 Gothenburg, Sweden; michael.kann@gu.se; 4 Department of Clinical Microbiology, Sahlgrenska University Hospital, 41345 Gothenburg, Sweden

**Keywords:** parvoviruses, nucleus, imaging of viral interactions and dynamics, analysis of protein–protein interactions, analysis of virus–chromatin interactions

## Abstract

Parvoviruses are small single-stranded (ss) DNA viruses, which replicate in the nucleoplasm and affect both the structure and function of the nucleus. The nuclear stage of the parvovirus life cycle starts at the nuclear entry of incoming capsids and culminates in the successful passage of progeny capsids out of the nucleus. In this review, we will present past, current, and future microscopy and biochemical techniques and demonstrate their potential in revealing the dynamics and molecular interactions in the intranuclear processes of parvovirus infection. In particular, a number of advanced techniques will be presented for the detection of infection-induced changes, such as DNA modification and damage, as well as protein–chromatin interactions.

## 1. Introduction

Parvoviruses are not only significant pathogens causing diseases in humans and animals but also promising candidates in gene therapy, in oncolytic therapy, in vaccine development, and as passive immunization vectors [1,2,3,4,5,6,7]. Compared to some other viruses that only need a few viral particles for infection, parvoviruses are extremely inefficient. In infection and disease development, this incapability is compensated by high replication. Finding new ways to treat parvoviral diseases and to facilitate the development of parvovirus-based therapies requires deepening the understanding of infection and propagation in their host cells. 

Although parvoviruses and their infection have been extensively studied throughout the past decades, there is still a lack of molecular level understanding of the virus–host cell interactions. Due to their low particles to infectious unit ratio, the identification and tracking of virus-induced events, which contribute to viral propagation, is a key challenge. Furthermore, the small size of parvovirus (~20 nm in diameter) hinders the attachment of fluorescent probes, which limits capsid detection by single-virus tracking. 

Parvoviruses are divided into two classes: autonomous parvoviruses, such as canine parvovirus (CPV), minute virus of mice (MVM), and rat parvovirus (H-1PV), and dependoparvoviruses, such as adeno-associated viruses (AAV), which require coinfection with either adenoviruses or herpes simplex virus in their late stages of infection [8]. Parvoviruses are composed of two to three capsid proteins (viral proteins, VPs; VP1, 2, and 3). They enclose a c. 5 kb-long ssDNA genome, which consists of two overlapping open reading frames. The expression is controlled by two promoters, the early P4 and late P38. The former guides the expression of viral nonstructural proteins 1 and 2 (NS1 and NS2), while the latter controls the expression of capsid proteins [9,10,11]. In the infectious virion, which has a diameter of 18–26 nm, the genome is covalently bound to the NS1 (Rep78 in AAV) protein [12,13,14,15]. This protein is cytotoxic and has central roles in viral replication attributed to its helicase, endonuclease, ATPase, and site-specific DNA-binding activities [16,17]. NS2 plays a role in viral replication [12,18], development of viral replication centres [19], viral mRNA translation [20], and the assembly [21] and nuclear egress of capsids [22,23,24,25,26]. In gene therapy, which is mostly based on AAV, the single-stranded genome is replaced by a double-stranded self-complementary genome, which does not allow replication [15]. 

After the cellular entry and cytoplasmic release, parvoviral capsids enter the nucleus through the nuclear pore complexes (NPCs) and/or via disruption of the nuclear envelope (NE) [27,28,29,30,31,32,33,34]. The VP1 capsid protein bears nuclear localization signals (NLSs) within its VP1-unique region in the N-terminal domain [35,36,37,38,39,40,41], which are thought to allow nuclear import by interaction with nuclear transport factors of the importin family [30,42,43]. In assembled capsids, this domain is hidden. 

Once arriving in the nucleus, the genome replicates via a rolling circle mechanism, during which the genome concatemer is cleaved to monomers by NS1 [44]. The gene expression of parvoviruses is coupled to the S-phase of the cell cycle, and it leads to the formation of distinct replication centre foci where viral gene transcription and productive replication occur [19,45,46]. As the infection proceeds, the replication centres expand [27,28,47], which is accompanied by changes in the cellular chromatin structure and chromatin marginalization to the nuclear periphery at later stages of infection [45,47]. Besides the dramatic morphological changes, parvovirus infections are known to induce substantial damage to the host DNA [48,49,50], and MVM replication centres have been shown to associate with the sites of cellular DNA damage [51,52]. This allows the virus to recruit cellular DNA replication and DNA damage response proteins, which promote viral replication and gene expression [45,49,53]. NS1 of MVM is responsible for nicking the host DNA, which subsequently results in S phase cell cycle arrest [54]. However, during human parvovirus B19 (B19V) infection, a G2/M arrest is induced by the NS1 protein through a p53-independent pathway, which does not depend on the DNA damage response [50]. In addition to evoking disturbances in the cell cycle, parvoviruses are known to cause apoptosis of the infected cells, another hallmark of DNA damage [55,56]. 

These nuclear changes are followed by progeny capsid assembly in the nucleus, which is combined with the encapsidation of viral genomes covalently bound to NS1. The progeny virions leave the cell by lysis, probably after export from the nucleus [57,58,59,60]. This lytic viral release, in conjunction with the S-phase-dependent replication, enables the use of autonomous parvoviruses in oncotherapy for the destruction of rapidly dividing cancer cells. [61].

## 2. Imaging of Viral Interactions and Dynamics in the Cytoplasm and Nucleus

**Figure 1:**
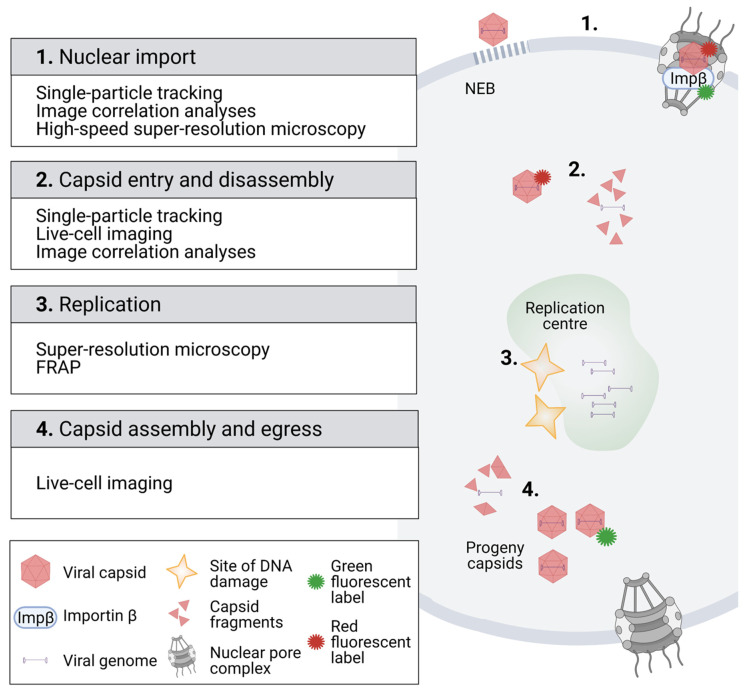
Imaging of viruses in the nucleus of infected cells.

To date, a broad variety of microscopy-based imaging and spectroscopy applications have enlightened the steps in the early infection of several parvoviruses (Figure 1). Upon nuclear import, CPV can pass the NE [27,28,62,63], which was confirmed by single-particle tracking analyses of fluorophore-labelled AAV capsids (Figure 1, boxes 1 and 2) [64]. Similar analyses have also been used to study the receptor binding of canine parvovirus [65,66] as well as the cytoplasmic trafficking [67] and nuclear import of AAV [27,28,64,68]. 

The schematic represents the fluorescent microscopy methodology for the imaging of the parvoviral life cycle in the nuclear region. (1) Analysis of fluorescent virus particle dynamics by single-particle tracking and high-speed super-resolution microscopy verified the import of viral capsids through the nuclear pore complex. Image correlation analysis using the pair correlation function (pCF) revealed the importin β-mediated nuclear transport of capsids. Confocal microscopy combined with EM characterized an alternative nuclear entry pathway for parvoviruses through virus-induced nuclear envelope ruptures. (2) Tracking of fluorescent capsids after their nuclear entry demonstrated that they moved by diffusion in the nucleoplasm. Furthermore, image correlation using the autocorrelation function (ACF) indicated that the capsids were disintegrated after their nuclear import. (3) Super-resolution microscopy analysis indicated that viral replication centres were located close to sites of cellular DNA damage. Fluorescence recovery after photobleaching (FRAP) studies showed that infection affected the diffusion of nuclear proteins, such as transcription-associated proteins. (4) Fluorescent tagging of progeny capsids (green) has allowed for analyses of capsid dynamics in living cells. Images were created with BioRender.com.

Imaging of autonomous parvovirus capsids has partially been hampered by the limited possibilities to express recombinant viruses that contain fluorescent proteins, as the enlarged genome size leads to poor viral genome packaging. Therefore, little is known about virus–nucleus interactions following the assembly of viral capsid. However, AAV-2 studies have shown that large peptides can be inserted into the VP2 protein with a minimal effect on viral assembly or infectivity [69]. This has allowed the creation of fluorescent protein-tagged AAV particles for live cell analysis of intranuclear dynamics [70]. The loop regions of AAV capsid proteins exposed to the capsid surface have been used for the insertion of shorter peptides, which enables the labelling of viral particles with a fluorescent dye [71,72].

Tracking of individual viruses is a powerful tool to examine the mechanisms of their intracellular transport, and it is straightforward, for example, to conclude whether the motion is directed or random diffusion. For active processes, such as transport along microtubules, the dynamics can be deduced from a low number of particles. However, insight into the parvoviral life cycle has revealed the diffusive dynamics of events. For example, following of the trajectories of Cy5-labelled AAV capsids in the cytoplasm and nucleus showed that the majority of capsids move by regular diffusion, but a smaller fraction of the capsids exhibits anomalous subdiffusion [64]. The analysis of a small number of randomly moving diffusing particles is challenging, but when the motions of typically hundreds or thousands of particles are averaged, their movement can be characterized. The mean squared displacement (MSD) of the particles follows the law MSD=2dDt, where D is the diffusion coefficient of the particle, d is the dimensionality of the motion, and t is the time. Measuring the MSD allows for the determination of the particle diffusion coefficient, which can then be further connected to the particle radius r, temperature T, and viscosity η of the medium by the Stokes–Einstein equation:

$$D=\frac{k_{B}T}{6\pi \eta r}.$$

Recently, image correlation spectroscopy has been used to verify the nuclear capsid import and intranuclear disassembly of capsids in living cells (Figure 1, boxes 1 and 2) [30]. Image correlation methods are based on the principles of fluorescence correlation spectroscopy (FCS), which measures fluctuations of fluorescence intensity in a small volume by using the focused excitation laser beam. The recorded fluctuations in photon counts, collected as a time series, are used to calculate the time autocorrelation function (ACF) to resolve the dynamics of fluorescently tagged proteins. The ACF represents the correlation of the fluorescent signal between the starting time point (t = t_0_) and following time points (t = t_0_ + ∆t) of the experiment, thus yielding information on fluorescent molecule diffusion time in the focal spot. In parvovirus studies, the ACF calculated for a time series of laser scanning microscopy images containing temporal information of the intensity fluctuations and spatial distribution maps of the fluorescent viral particles has enabled the analysis of fast and slow diffusion, or even immobile viral particles [30].

To obtain more information about the possible directed movement of fluorescent particles, pair correlation function (pCF) analysis can also be used. The pCF measures the correlation over time and space and thus can distinguish directed movement or obstacles to diffusion. In parvovirus studies, pCF revealed a positive correlation between pixels across the NE within an image series, thereby demonstrating the nuclear import of capsid through the NE [30,73,74,75]. In addition, pCF analysis detected a spatiotemporal correlation between the fluorescent viral capsid and importin β, suggesting that importin β mediates capsid translocation through the nuclear pore complex [30]. An alternative or parallel existing nuclear entry pathway has been derived from studies using fluorescence and electron microscopy. The experiments have demonstrated that the NE undergoes substantial damage at early times during parvovirus H1, CPV, and AAV2 infection, indicating an NPC-independent nuclear entry of capsids [31,33].

The theoretical nuclear diffusion coefficient of capsids obtained from the Stokes–Einstein law, assuming that the viscosity of the nucleoplasm is approximately four times higher than in water [76,77], is in the order of 10 µm^2^/s. This is in accordance with the experimental finding of 5 µm^2^/s obtained for the mobile population of virus-like particles of parvovirus [30,47]. In the cellular scale, this is a relatively fast diffusion rate, and it means that on average, the virus particles are able to diffuse a 10 µm distance in a time scale of a few seconds, when not restricted by physical barriers or by interactions. 

Studies of nucleoplasmic capsid diffusion coefficients by ACF, which improved temporal resolution from the millisecond to microsecond scale, have revealed distinct diffusion dynamics for intact capsids and potential capsid fragments, suggesting that capsids are disintegrated in the nucleoplasm after their import [30]. The detailed mechanisms by which the viral genome is released into the nucleoplasm remain to be determined. However, fluorescence microscopy analyses have shown that capsids are already modified prior to nuclear import and nuclear disassembly when VP1 N-terminus is exposed during the endocytic entry [41,78,79,80]. According to immunoprecipitation analyses, B19V capsid uncoating is enhanced by cytoplasmic divalent cations [81]. Previously published studies have demonstrated that at least for MVM, the nuclear release of DNA occurs without a complete disassembly of the capsids [78,82,83,84,85]. In summary, it can be concluded that parvoviral capsids enter the nucleus either via NPC or by passing through transient holes in the NE, which allow the entry of intact capsids. Intact capsids entering the nucleus may undergo structural change which leads to viral genome release at some distance from the NE [30,86].

As outlined before, progressing parvovirus infection leads to the development of viral replication centres [46,87] and relocation of host chromatin to the nuclear periphery [45,47,48,49,88]. Recently, super-resolution microscopy has demonstrated that viral replication centres originate close to DNA damage sites (Figure 1, box 3) [52]. The introduction of photobleaching experiments in the analyses of intranuclear mobility and kinetics of viral and cellular proteins has allowed a better monitoring of nuclear changes upon parvoviral infection (Figure 1, box 3). In these studies, a high-intensity laser is used to photobleach the fluorescence of a fluorescent molecule, typically a fluorescent fusion protein, from a defined area of the cell. In fluorescence recovery, after photobleaching (FRAP), a region of interest is bleached, and the recovery of fluorescence in the bleached region is measured. The rate of fluorescence recovery is determined by the exchange of fluorescent molecules between the bleached region and the surrounding unbleached area, thereby allowing the analysis of protein dynamics and interactions. In fluorescence loss in photobleaching (FLIP), an area of the cell is continuously photobleached with laser pulses, and images taken between the pulses measure the response in the entire pool of fluorescent molecules. Similar to FRAP, the rate of fluorescence loss is related to the mobility of the fluorescent molecules.

In CPV infection, FRAP experiments (Figure 1, box 3) have revealed that the dynamics of transcription-associated protein change during infection [89] and further demonstrated that infection leads to an increased protein mobility in the nucleoplasm, which potentially alters protein–protein and protein–DNA binding reactions during viral replication [47]. Additionally, FRAP has been used to study the kinetics of NS1-EYFP in noninfected cell nuclei. The results have shown that NS1-EYFP mobility is not consistent with free diffusion and suggested transient binding to nuclear components [90]. Shown by FLIP, the nucleocytoplasmic shuttling of NS1-EYFP has been discovered [90].

Further central questions in the late stages of the nuclear life cycle of parvoviruses, such as capsid assembly and nuclear egress, have been addressed using fluorescent microscopy of immunostained cells. These studies, in combination with biochemical characterizations, showed that MVM capsids assemble in the nucleus from VP1/VP2 trimers [60,91], and these trimers expose a structured nuclear localization motif [58]. For AAV-2, the subcellular localization of capsid assembly to nucleoli was identified with immunofluorescence and in situ hybridization microscopy techniques. Viral genome sequence analysis and mutational studies revealed that the capsid assembly is mediated by the viral assembly associated protein (AAP) [92,93]. Moreover, X-ray crystallography and cryo-EM analyses of MVM capsids demonstrated that viral DNA is packed through a fivefold packaging channel [94,95]. Studies have also revealed that MVM capsids leave the nucleus prior to cell lysis and NE breakdown [96], suggesting that capsids have to exit the nucleus through the NPCs [22,23]. A similar combination of techniques was used to show that MVM capsids egress the nucleus dependent upon chromosomal region maintenance 1 (CRM1, also known as exportin 1) protein [96], which is a nuclear export factor for various proteins and different cellular RNAs (snRNA, rRNA, some mRNAs) [97]. Notably, the nuclear exit was limited to genome-containing capsids phosphorylated in the unordered domain of VP2, while empty capsids exhibited nuclear accumulation [96]. By combining classical immunofluorescence microscopy with surface plasmon resonance spectroscopy, it has been shown that the CRM1-dependent nuclear export of MVM capsids is mediated by the supraphysiological NES in NS2 [22].

## 3. Screening and Validation of Protein–Protein Interactions

**Figure 2:**
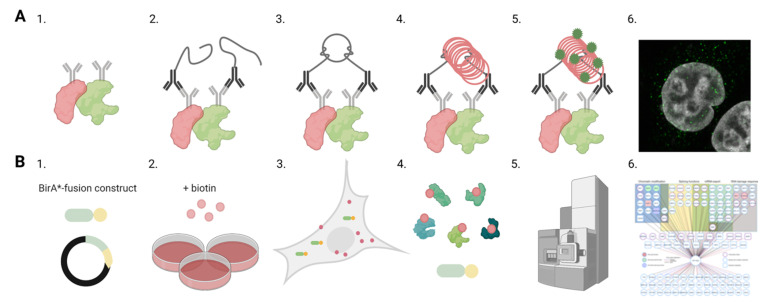
Analyses of protein–protein interactions in infection. Schematic overviews of proximity ligation assay (PLA) and proximity-dependent biotin identification (BioID) methods to identify and localize interactions between viral and host proteins. (**A**) The schematic representation of PLA assay. (1) Primary antibodies are used to target proteins of interest shown in red and green. (2) Secondary antibodies with PLA oligonucleotide probes bind to the primary antibodies. (3) Closely located PLA probes are ligated together, and (4) the formed circular DNA is amplified. (5) The amplified DNA (red) is labelled by fluorescent probes (green). (6) Confocal microscopy image shows the intracellular distribution of the PLA signals (green). Nuclei were stained with DAPI (grey). (**B**) Outlines of the BioID workflow. (1) Transfection of cells with BirA*-viral protein-fusion constructs and the generation of a stable inducible cell line. (2) Addition of biotin to the culture media and viruses if infection is required. (3) Cell culture period during which biotin ligase activity of BirA* fusion protein induces proximity-dependent biotinylation of neighbouring endogenous and viral proteins. (4) Cell lysis and the streptavidin-affinity purification of biotinylated proteins from cell lysates. (5) Mass spectroscopy and analyses of protein associations. (6) Interaction network indicating interaction partners of viral protein and biological processes involved. Images were created with BioRender.com.

The nuclear import of intact parvovirus capsids is not limited by the NPC diameter, which is able to transport particles with a diameter of ~39 nm [98]. There is accumulating evidence that the nuclear entry of the parvovirus capsid depends on the host machinery for nuclear import, requiring coordinated interaction with different host proteins. Earlier studies have shown that the capsid proteins of MVM and CPV, in addition to AAV capsids, have basic regions containing NLSs or a structured nuclear localization motif in their capsid proteins. [35,36,37,38,39,40,41,60,79] During endocytic entry, the acidification of capsid leads to NLS exposure, and after reaching the cytoplasm, this would thus allow the attachment of nuclear import factors. Studies including coimmunoprecipitation assays (Co-IP) have verified that CPV and AAV2 capsids interact with Imp β [42,99]. However, these assays elucidate neither the localization of the interaction in the cell environment nor the phase of the infection. The proximity ligation assay (PLA) has allowed comprehensive imaging and quantitation of interactions within the host cell. This antibody-based technique enables the detection of two proteins that are in close proximity to each other (~40 nm) [100]. Therefore, PLA is capable of visualizing protein–protein interactions beyond the diffraction limit (Figure 2A). For CPV, in situ proximity ligation analysis, combined with confocal microscopy and image analysis, has demonstrated that capsids are able to recruit cytoplasmic Imp β for nuclear transport [42]. Coimmunoprecipitation analyses have indicated that entering H-1PV and AAV2 capsids interact with nucleoporins, which are proteins of the NPC [31].

Knowledge of viral protein interactions with cellular proteins is essential for understanding the intranuclear processes such as viral replication, capsid assembly, and nuclear egress. Affinity purification-mass spectrometry proteomics approaches have been traditionally used to analyse protein–protein interactions in infection [101,102,103]. Recently, many new screening methods have been generated to recognize protein–protein associations [104,105,106]. One of the methods is the proximity-dependent biotin identification (BioID) assay combined with mass spectrometry [107,108,109] (Figure 2B). BioID is a proximity-tagging method that utilizes a fusion of promiscuous biotin ligase, BirA, to a protein of interest to identify protein–protein associations and proximate proteins. The working radius for biotinylation via BirA is 10–40 nm, depending on the used application. Mass spectrometry-based proteomics applications such as BioID are able to recognize highly transient protein–protein interactions during the viral lifecycle. BioID studies of parvovirus human bocavirus 1 (HBoV1) have revealed interaction between viral nuclear protein 1 (NP1) and factors mediating nuclear import and mRNA processing [110]. A BioID analysis of AAV2 Rep proteins has revealed their association with cellular proteins, such as the transcriptional corepressor KAP1, which assist the viral genome in resisting epigenetic silencing, thereby allowing the lytic replication of AAV [111]. BioID has also been used to recognize interactions between viral proteins and DNA damage-related proteins. BioID has revealed an AAV Rep protein interaction with the Mre11 part of the MRN complex, an important initiator of the AMT response [111]. Overall, BioID has allowed for identifying associations of the viral protein of interest in a wide variety of nuclear processes, which, for CPV NS2, include DNA damage response and chromatin modification [112].

## 4. Detection of DNA Damage, DNA Repair, and Virus–DNA Interactions

**Figure 3:**
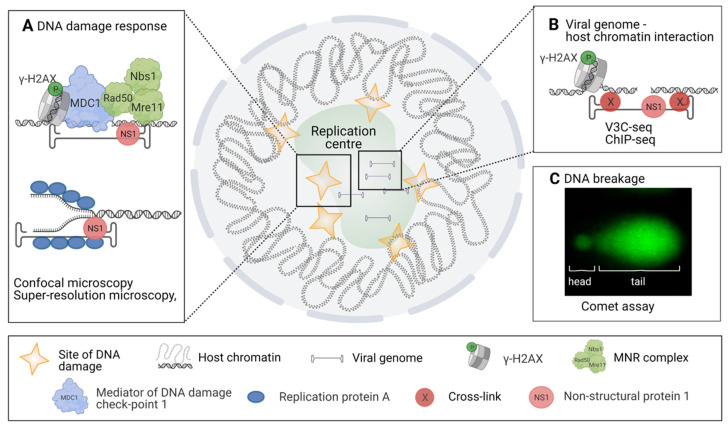
Approaches revealing virus-induced DNA damage. The schematic diagram of diverse methods for the analyses of DNA damage response (DDR), viral and host DNA interactions, and DNA damage in infection. (**A**) Analyses of ATM and ATR-mediated DNA damage signalling pathways by confocal and super-resolution microscopy, ATM-mediated cellular response to DNA damage functions through phosphorylation of proteins related to DNA damage and DNA damage repair such as γ-H2AX, MDC1, Rad50, Nbs1, and Mre11. In MVM infection these proteins are found in replication start sites together with viral NS1. In parvovirus-infected cells, the ATR-mediated response depends on RPA and viral NS1 interaction. (**B**) Elucidation of interactions between viral genome and host cell chromatin by using high-throughput viral chromosome conformation capture sequencing assay (V3C-seq). Moreover, association of DNA damage site MVM genomes has been shown by ChIP-seq. This analysis has been used to verify the association between NS1-mediated viral genome replication and DDR. (**C**) Studies of host cell chromatin disintegration by comet assay. Images were created with BioRender.com.

Progression of parvovirus infection depends upon the induction of a cell cycle arrest and cell lysis. It leads to the activation of DNA damage response (DDR) [19,45], which promotes the infection and viral reproduction [113,114]. Ataxia telangiectasia and Rad3(ATR)-mediated DDR activation is linked to replication fork stalling, whereas the activation of the Ataxia-telangiectasia mutated (ATM)-mediated route is the initial response to a double-stranded DNA break (DSB) [115,116]. The activation of the ATR route has been observed for MVM, B19, and HBoV1 [51,111,112], and the ATM route for MVM, HBoV1, and AAV [45,117,118,119] (Figure 3A). Recognition of DNA damage induces the recruitment of proteins responsible for DNA damage repair to the site of the damage. During parvovirus infection, the emergence of DNA damage can be observed either indirectly by the accumulation of DDR proteins to the damage site or by observing the formation of actual DNA breakages. MVM infection has been shown to cause accumulation of proteins of the ATM signalling route (e.g., phosphorylated H2AX (γ-H2AX), Nbs1, RPA32, Chk2, p53, MDC1, MRN) to the replication start sites together with the viral replication protein NS1 [19,45]. During viral replication, at least newly synthesized viral DNA is bound to RPA, a known activator of ATR [120]. However, in MVM infection, this does not lead to the full activation of the ATR response since checkpoint kinase1 (Chk1) is not activated [49,51] (Figure 3A).

Recently, a high-throughput viral chromosome conformation capture sequencing assay (V3C-seq) has been applied to study the association of MVM viral genomes with host chromatin [121] (Figure 3C). V3C-seq is based on the chromosome conformation capture sequencing technology (3C-seq) [122] used to study chromosome arrangement in the nucleus by crosslinking the sites of genomic associations and identifying these regions with sequencing. 3C-seq studies have revealed that MVM genomes become associated with DNA damage sites during early stages of infection [121]. These sites of DNA damage with associated viral genomes increase as the infection proceeds. Nuclear localization of this association was further verified with fluorescent in situ hybridization (FISH) and super-resolution stochastic optical reconstruction microscopy (STORM). The introduction of externally induced DNA damage sites with laser irradiation or with CRISPR-Cas9 to a specific genomic locus resulted in parvoviral genome association with these regions. V3C-seq analyses have also revealed that the viral genome association sites and DNA damage sites overlap with self-interacting genetic regions, also known as topologically associating domains (TADs) [52]. Recently, it has been shown that the localization of viral genomes to the DNA damage sites is mediated by viral NS1 [121].

Classical DNA damage analyses in viral infection are qPCR or agarose gel electrophoresis, which do not allow investigations on the single-cell level. This obstacle was solved by comet assay—also known as single-cell gel electrophoresis—which is a sensitive, quantitative, and relatively simple imaging-based method to observe DNA breakages (Figure 3C) [123,124,125]. Scraped or trypsinized cells are cast into low-density agarose gel and lysed, after which the remaining nucleoids are placed in an electric field and stained. DNA lesions, both single and double stranded, result in a relaxation of DNA supercoiling. The relaxed DNA loops migrate towards the positively charged pole during electrophoresis, forming the characteristic comet tail pattern. The relative DNA content in the comet tail versus the head thus reflects the number of DNA lesions. Unlike the various DDR pathway markers, which might be activated in response to viral genomes or proteins [126], this method relies on the physical properties of damaged host DNA. Comet assay studies and ChIP-seq analysis have demonstrated that MVM infection causes host DNA damage, which increases as the infection proceeds [52]. In contrast, the comet assay has revealed no significant DNA damage in cells infected by the bocavirus minute virus of canine [127], nor in cells infected by human B19V [127]. The potential nucleolytic activity of parvoviral NS1 protein against host DNA has been investigated in expression studies for HBoV1 [117] and human B19V [127], but these studies did not find significant host DNA damage in NS1-expressing cells.

To benefit from host cell responses such as the DDR, viral proteins or viral genomes are required to interact directly with DNA or DNA-modifying proteins. The interactions of cellular DNA-binding proteins and viral proteins with host chromatin and viral genomes in MVM and CPV infections have been studied by ChIP-seq methods [52,88,121]. These studies have shown the acetylation of histones bound to CPV genome and MVM genome association with cellular γ-H2AX sites and the viral NS1 protein [52,88,121] (Figure 3B). Furthermore, the studies of the genomic reactivation of latent AAV genome by ChIP and ChIP coupled to qPCR have revealed the mechanism by which cellular proteins induce viral genome repression [111].

## 5. Recent Methods for Future Studies of Parvovirus–Nucleus Interactions

Despite of decades of research, many detailed mechanisms of virus–host interactions are not well understood, and many new observations raise further questions, requiring the use of newly developed techniques. Next-generation sequencing (NGS) and fluorescence imaging technologies are currently advancing rapidly [128,129], offering excellent opportunities for detailed analysis of infection-induced changes in the host chromatin organization and high-resolution imaging of parvovirus infection. For example, these methods combined with spatial transcriptomics allow analyses of the spatial heterogeneity of the gene expression within the sample [130,131,132,133].

NGS is a modern sequencing methodology where massive parallel sequencing is used to map the sequences of millions of small DNA fragments. Bioinformatics is then used to combine the acquired sequencing data, which can be then compared to reference genome(s). Various approaches allow for obtaining information about expressed genes [134], genome accessibility [135], binding regions of different DNA interacting proteins [136,137,138], or chromatin–chromatin interactions and organization [139]. As an example, the assay for transposase-accessible chromatin with sequencing (ATAC-seq) is based on hyperactive Tn5 transposase mutants [135]. In this assay, the hyperactive Tn5 is used to tagment the accessible chromatin by conjugating short and specific DNA oligomers into the accessible regions. These regions of the genome are then isolated and sequenced, yielding a high-resolution map of the accessible regions of the genome. Thus, ATAC-seq has great potential in studies on how parvoviral infection changes the host cell chromatin organization or in studies of viral genome packaging or release. This is exemplified by recent results showing that baculovirus infection induces significant changes in the organization of host genome, such as an increase in chromatin accessibility, relocation close to the NE, and nucleosome disassembly [140]. Moreover, ATAC-seq analysis of Epstein–Barr virus (EBV), a member of the herpesvirus family, has demonstrated that B cell chromatin undergoes significant remodelling during infection, which leads to the regulation of cell cycle, apoptosis pathways, and interferon regulatory factors [141]. Another example of a similar DNA-tagging method is DNA adenine methyltransferase identification (DamID)-sequencing [142]. Here, DNA adenine methyltransferase (Dam) is fused to a protein of interest, and this fusion protein is expressed in cells. The Dam enzyme recognizes DNA sequence GATC and methylates the adenine in the close vicinity of the fusion protein. These methylated regions of chromatin can then be sequenced and mapped. Thus, these sequences correspond to the chromatin that has been in close vicinity to the expressed fusion protein. This DamID-seq has been used to map the chromatin interacting with the nuclear lamina and lamina-associated domains [143]. In addition to sequencing, both ATAC-seq and DamID-seq can be combined with high-resolution fluorescence imaging. In the case of ATAC-seq, fluorescent oligomers are used together with hyperactive Tn5, and therefore, the tagmented and accessible chromatin can be visualized by fluorescence microscopy. This ATAC-see method [144] allows imaging the accessible chromatin regions and would be directly applicable to parvoviral studies regarding host cell chromatin or viral genome organization. DamID can be used together with methylated DNA-recognizing fluorescent ^m6^A-tracer fusion protein. ^m6^A-tracer binds to the GATC sequence when adenine is methylated by Dam methylase. By fusing ^m6^A-tracer to a fluorescent protein, the fluorescent signal localizes to the methylated DNA [145]. The great advantage of the DamID ^m6^A-tracer system is the possibility to use it in living cells. Thus, one can follow the chromatin dynamics by live cell microscopy. We envision that the system could be used to follow parvovirus infection-induced dynamic reorganization of the host genome.

Imaging and sequencing approaches are directly combined in spatial transcriptomics, where transcriptomes are resolved by high resolution microscopy or by capturing, so that spatial information about the location is also recorded. In microscopy-based spatially resolved transcriptomics or genomics, the different RNA and DNA species are labelled via sequential fluorescence in situ *hybridization* and barcoding. This approach offers the highest resolution, and recently, the imaging of 3660 chromosomal loci together with 17 chromatin marks in single cells has been reported [146].

## 6. Concluding Remarks

Conventional confocal microscopy approaches, including the imaging of fluorescent viral capsids and proteins and their interplay with cellular components within the host cell, have been successfully used in parvovirus studies. The development of live cell imaging and super-resolution microscopy, combined with image data analysis, together with the development of new screening tools for analyses of protein–protein and DNA–protein interactions, has further enhanced our understanding of virus–nucleus interactions and the nuclear dynamics of infection. In the near future, combining fluorescence data and ultrastructural information from electron micrographs will allow answering detailed questions regarding the mechanisms of intranuclear events in viral infection. Moreover, the advances in super-resolution microscopy applications will enable us to probe cell–virus interactions and dynamics in previously unattainable detail.

## Data Availability

Data available in a publicly accessible repository.
